# Comparing risk factors of HIV among *hijra* sex workers in Larkana and other cities of Pakistan: an analytical cross sectional study

**DOI:** 10.1186/1471-2458-12-279

**Published:** 2012-04-10

**Authors:** Arshad Altaf, Aysha Zahidie, Ajmal Agha

**Affiliations:** 1 Canada-Pakistan HIV/AIDS Surveillance Project, Sindh AIDS Control Program, Karachi, Pakistan; 2 Department of Community Health Sciences, The Aga Khan University, Karachi, Pakistan

**Keywords:** Commercial sex workers, HIV prevalence, Urban Sindh

## Abstract

**Background:**

In 2005, Pakistan was first labeled as a country with concentrated epidemic of Human Immunodeficiency Virus (HIV). This was revealed through second generation surveillance conducted by HIV/AIDS Surveillance Project (HASP). While injection drug users (IDUs) were driving the epidemic, subsequent surveys showed that *Hijra* (transgender) sex workers (HSWs) were emerging as the second most vulnerable group with an average national prevalence of 6.4%. An exceptionally high prevalence (27.6%) was found in Larkana, which is a small town on the right bank of river Indus near the ruins of Mohenjo-Daro in the province of Sindh. This paper presents the risk factors associated with high prevalence of HIV among HSWs in Larkana as compared to other cities of the country.

**Methods:**

Data were extracted for secondary analysis from 2008 Integrated behavioral and biological survey (IBBS) to compare HSWs living in Larkana with those living in other cities including Karachi and Hyderabad in Sindh; Lahore and Faisalabad in Punjab; and Peshawar in Khyber Pakhtunkhwa provinces. After descriptive analysis, univariate and multivariate analyses were performed to identify risk factors. P value of 0.25 or less was used to include factors in multivariate analysis.

**Results:**

We compared 199 HSWs from Larkana with 420 HSWs from other cities. The average age of HSWs in Larkana was 26.42 (±5.4) years. Majority were Sindhi speaking (80%), uneducated (68%) and unmarried (97%). In univariate analysis, factors associated with higher prevalence of HIV in Larkana included younger age i.e. 20–24 years (OR: 5.8, CI: 2.809–12.15), being unmarried (OR: 2.4, CI: 1.0–5.7), sex work as the only mode of income (OR: 5.5, CI: 3.70–8.2) and longer duration of being involved in sex work 5–10 years (OR: 3.3, CI: 1.7–6.12). In multivariate logistic regression the HSWs from Larkana were more likely to lack knowledge regarding preventive measures against HIV (OR 11.9, CI: 3.4–41.08) and were more prone to use of alcohol during anal intercourse (OR: 6.3, CI: 2.77–17.797).

**Conclusion:**

Outreach programs focusing on safer sexual practices and VCT are urgently needed to address the upsurge of HIV among HSWs in Larkana.

## Background

Injection drug users (IDUs) have been driving the human immunodeficiency virus (HIV) epidemic in Pakistan since it has progressed from low to concentrated level of epidemic [1]. The other high risk group rapidly emerging as most vulnerable to HIV is male sex workers and among them *Hijra* (transgender) sex workers (HSWs) are notably driving the epidemic to the general population [2]. In 2008, the national prevalence of HIV was reported to be 21% (95% CI: 19.4%, 22.3) among IDUs and in the same year the infection among HSWs was found to be 6.4% (95% CI: 5.0%, 7.7%) [3].

*Hijras* have a long recorded history in the Indian subcontinent, from ancient times, as suggested by the Kama Sutra period onwards. This history attributes a number of well-known roles for *Hijras* within the sub continental cultures. In South Asia, many *Hijras* live in well-defined, organized, all-*Hijra* communities, led by a Guru (group leader). These communities have sustained themselves over generations by "adopting young boys who are rejected by, or run away from their family of origin [4].

In Pakistan transgenders have been around for a long time. They used to earn their living by singing and dancing at births, weddings and festivals. With the passage of time their social acceptance in the Pakistani society declined which forced them towards begging on streets and subsequent commercial sex. Larkana is among the various urban centers of Pakistan where HSWs colonize. It is a small rice growing town in rural Sindh situated some 440 kilometers northwest of Karachi. According to the Population Welfare Department, the population of this town is approximately 450,000, mostly comprising of Sindhi speaking people. Larkana came into lime light in 2004 when first outbreak of HIV was documented there and initially 17 IDUs were found to be HIV positive. Later on, this number reached 45 [5]. The mapping study, conducted in 2007, estimated that there were also 600 HSWs scattered at 117 different spots of the town called *Deras*[6]. In Round III of national surveillance the prevalence of HIV infection among HSWs of Larkana was found to be a staggering figure of 27.6% (21.4, 33.9%) while in Karachi, a mega city with much higher number of HSWs the prevalence was 3.6% (1.2, 6.1) and at the same time in Hyderabad, no HSW was found to be HIV positive [3] (Table 1). Emmanuel at al. have estimated that there were 4.9 HSWs per 1000 adult men in Larkana [7], showing one of the highest proportion in the country as compared to Karachi, where there are 1.7 HSWs per 1000 adult men. Larkana is unique in some ways because this city has a functioning brothel where clients visit FSWs (female sex workers) in day time as well. In the middle of the town lies the Station Road famous because of its close proximity to the city’s railway station. This road has numerous hotels, motels, tea stalls and shops and it remains lively throughout the day and night time. On this road are also located a number of small bed and bathroom type motels called “*musafirkhanas*”. These motels are popular for male commercial sex. In these *musafirkhanas* HSWs and their pimp hire a number of rooms and clients visit them throughout the day for receptive anal sex. HSWs in Larkana entertain an average of 3.3 clients per day. In 2008 the average age of HSWs in Larkana was found to be 26.4 years, age of initiation of sex 13.6 years and duration in sex work 12.8 years. Less than 50% reported using a condom in their last paid sexual encounter [8].

**Table 1:** Prevalence of HIV among HSWs in Larkana as compared to other cities

**City**	**Tested**	**Positive**	**Prevalence**
Karachi	222	8	3.6%
Hyderabad	198	0	0
Larkana	199	55	27.6%
Faisalabad	200	5	2.5%
Lahore	201	5	2.5%
Peshawar	161	2	1.2%
**Total**	1181	75	6.4%

It establishes the presence of a very active and mobile group of HSWs in Larkana. These HSWs who exchange sex for money are at an extremely high risk of HIV/AIDS and may act as bridging population between several at-risk groups. It is because they may get engaged in unsafe sexual behaviors with their clients, most of whom are gay or bisexual men, but who may also have female sex partners [9].

To address the links between HIV and sex work, policies and programs must recognize the socio-geographic diversity and characteristic patterns of sex work amid various high risk groups along with the modes of interaction that takes place among them [10]. Limited national studies are available revealing sex behavior pattern and its linkage with HIV and STI among HSWs in Pakistan. In Round III (2008) behavioral and biological survey data on HSWs was collected using a structured questionnaire covering questions on socio-demographic information, knowledge related to HIV/AIDS and STIs and risk behaviors. The purpose of this study is to measure the prevalence of sexual behaviors known to facilitate HIV transmission among HSWs in Larkana comparing it with factors among HSWs in other cities of the country.

## Methods

The data for secondary analysis were derived from Round III of the yearly integrated behavioral and biological surveys (IBBS) which was jointly conducted by Canada-Pakistan HIV/AIDS Surveillance Project, National and Provincial AIDS Control Programs, Pakistan. Survey was conducted from March to June 2008. It included three major high risk groups (HRGs) for HIV/AIDS, including Injecting Drug Users (IDUs), male sex workers (MSWs) and *Hijra* sex workers (HSWs) in major cities of Sindh, Punjab and Khyber Pakhtunkhwa (KPK) (formerly NWFP) provinces. The cities included Karachi, Hyderabad and Larkana in Sindh; Lahore and Faisalabad in Punjab; and Peshawar in KPK. In the initial planning of surveillance round, Quetta in Baluchistan was also included, but was later suspended due to security issues. The selection of cities was based on the presence of multiple HRGs and the presence of a service delivery program (SDP) in the targeted city. Mapping results from 2006–07 were used to develop sampling frames required for recruiting study subjects.

### Sample size

In primary data set a total number of 1,186 HSWs were interviewed in aforementioned six cities. After data cleaning and elimination of forms with missing information, 619 participants were selected for secondary analysis.

### Sampling technique and recruitment of study participants

HSWs were recruited through Network Sampling in which *Deras/Gurus* were selected randomly from a list of *Gurus/Deras* available from previous mapping results. All HSWs in the selected *Deras* were recruited for the study. They were paid Rupees 300 (US$ 4) for their time and participation in this study.

### Sample selection

#### Inclusion criterion

Any transvestite/transsexual who undertakes sexual activity with a man in return of money or other financial benefits.

#### Exclusion criteria

Those with age under 15 yrs or over 45 yrs or not willing to participate in the study/unwilling to provide informed consent or who appears to be, in the interviewer’s judgment, incapable of understanding the information provided about the survey (e.g., due to intoxication, drug withdrawal, or the person is cognitively impaired, etc.) or a person who has already participated in the survey in the current round were excluded.

### Data collection procedure

#### Behavioral data

Behavioral data were gathered from HSWs using a structured questionnaire covering questions on socio-demographic information, knowledge related to HIV/AIDS and STIs and risk behavior.

#### Biological data

Upon completion of the interview, each participant was requested to provide a blood sample for HIV antibody testing. Test was conducted using the capillary “Dried Blood Spot” (DBS) methodology, chosen for its ease of collection, storage, shipping, and serological accuracy. All DBS specimens were first screened by the HIV Genetic Systems rLAV Enzyme immunoassay (ELISA/EIA) (Bio-Rad USA). Samples that tested positive were subsequently confirmed in duplicate by the Vironostika HIV Uni-Form II EIA (Biomeriux, The Netherlands). The Genetic Systems HIV-1 Western Blot (Bio-Rad USA) was used to confirm the status of any specimen found to be diagnostically indeterminate after EIA testing.

### Statistical analysis

Data were analyzed using Statistical Package for Social Sciences (SPSS) version 16. Descriptive analysis was done by calculating mean (+standard deviation) for continuous and proportions for categorical variables. Univariate analysis was conducted to compare characteristics of HSWs from Larkana with HSWs from other cities. The outcome variable was categorical i.e. HSWs living in Larkana or living in other cities (i.e. Karachi, Hyderabad, Faisalabad, Lahore and Peshawar). As all the independent variables were categorical, chi-square and Fisher’s exact tests were used to find out their association with outcome variables. To observe the independent effect of individual factors, the potential confounders were controlled by means of logistic regression analysis and odds ratios (OR) with 95% confidence interval (CIs). Variables with p value of 0.25 at univariate level were included in the MLR. The backward MLR model was used to determine the significant factors.

### Ethical review

The study was ethically reviewed and approved by ethical review committees of Health Canada in Canada and HOPE (NGO) in Pakistan.

## Results

A total number of 199 (HSWs) were interviewed in Larkana and their characteristics were compared with 420 HSWs living in other cities of the country. Mean age of HSWs living in Larkana was 26.42 ± 5.4. Sindhi (79.9%) was the predominant ethnicity. The majority of HSWs were uneducated (67.8%) and unmarried (96.5%). Among 80.4%, sex work was the only mode of income, 60.3% were living at some place other than their family homes (Table 2).

**Table 2:** Socio-demographic characteristics of HSWs living in Larkana and other cities

**Characteristics**	**Larkana(199)**	**Other cities(420)**
**Mean age**(±SD)	26.42(±5.4)	28.91(±6.1)
**Mother tongue**		
Urdu	1(0.5%)	215(51.1%)
Punjabi	23(11.6%)	81(19.5%)
Sindhi	159(79.9%)	52(12.2%)
Other	1(7%)	72(17.2%)
**Educational status**		
Educated	64 (32.2%)	152(36%)
Uneducated	135(67.8%)	268(64%)
**Marital status**		
Currently married	7(3.5%)	43(10.8%)
Unmarried	192(96.5%)	377(89.2%)
**Modes of income**		
Barber	1(0.5%)	15(3.7%)
Beggar	6(3%)	95(22.8%)
Dancer	17(8%)	125(30.1%)
Milkman	1(0.5%)	2(0.5%)
Painter	1(0.5%)	0(0%)
Shopkeeper	13(6.5%)	1(0.2%)
Only sex work	160(80.4%)	181(42.6%)
**Living arrangement**		
Family home	79(39.7%)	83(20%)
Dera	38(19.1%)	330(78%)
Hostel	7(3.5%)	2(0.5%)
Guest hotel	75(37.7%)	5(1.4%)
**Belong to this city**		
Yes	177(88.9%)	309(73.2%)
No	22(11.1%)	111(26.8%)

HSWs in Larkana encountered multiple partners in the month prior to data collection compared to HSWs in other cities (Table 3).

**Table 3:** Per month averages no. of clients among HSWs of Larkana and other cities

**CLIENTS**	**LARKANA**	**OTHER CITIES**
1–5Clients	143(71.9%)	401(96.2%)
5–10Clients	47(23.6%)	15(3.6%)
>10 Clients	9(4.5%)	1(0.2%)
**Total**	199	417

Table 4 shows results of univariate analysis with socio-demographic characteristics, risky sexual behaviors and knowledge regarding HIV prevention and its mode of spread as exposures against high HIV prevalence among HSWs living in Larkana or other cities as outcome.

**Table 4:** Univariate analysis comparing risk factors of HIV among Hijra Sex Workers in Larkana and other cities of Pakistan

Variables	**Larkana** **n = 199(32.1%)**	**Other Cities** **n = 420(67.9%)**	**OR**	**CI**
*Socio-demographic characteristics*				
**Age(in years)**				
19	28(4.5%)	23(3.7%)	5.84	2.81–12.15
20–24	47(7.6%)	71(11.5%)	3.17	1.73–5.83
25–29	68(11%)	131(21.2%)	2.49	1.42–4.38
30–34	36(5.8%)	99 (16%)	1.74	0.94–3.23
+35	20(3.2%)	96(15.5%)	1	–
**Educational status**				
Educated	64(10.4%)	152(36%)	1	–
Uneducated	135(21.9%)	268(64%)	1.21	0.85–1.74
**Marital status**				
Currently married	7(1.1%)	43(10.8%)	1	–
Unmarried	192(31%)	377(89.2%)	2.49	1.08–5.71
**Belong to current city of residence**				
No	22(3.6%)	309(73.2%)	1	–
Yes	177(28.6%)	111(26.8%)	2.99	1.83–4.90
**Mode of income**				
Other livelihood along with sex work	39 (6.3%)	241(38.9%)	1	–
Only sex work	160 (25.8%	179(28.9%)	5.52	3.70–8.23
**Current living with**				
· Others	13(6.53%)	2(0.5%)	1	–
· Alone	69(34%)	6(1.4%)	0.76	0.23–0.40
· Family or relatives	79(39%)	85(20%)	1.23	0.03–0.40
· Friends	38(19%)	332(78%)	0.21	0.12–0.38
**Duration in sex work**				
5 years or less	16(2.6%)	94(15.2%)	1	–
5.1–10 years	59(9.5%)	105(17%)	3.30	1.77–6.12
>10 years	124(20%)	221(35.7%)	3.29	1.85–5.85
**Age at first intercourse**				
8–15	151(24.4%)	272(44%)	1	–
15–20	45(7.3%)	144(23.3%)	0.31	0.11–2.78
<20	3(0.5%)	3(0.5%)	1.00	0.06–1.60
**Client contact through**				
Other	58(9.4%)	35(5.7%)	1	–
Pimp/Guru	40(6.5%)	105(17%)	0.23	0.13–0.40
Mobile phone	36(5.8%)	94(15.2%)	0.23	0.13–0.40
By roaming around	65(10.5%)	186(30)	0.21	0.12–0.35
**In the last 12 months, have you been**				
**away from this city for sex work?**				
No	116(18.8%)	290(46.9%)	1	–
Yes	83(13.4%)	129(20.9%)	1.60	1.13–2.28
				
**Condom used last time when had**				
**anal intercourse with a paying client**				
No	101(16.5%)	292(47.6%)	1	–
Yes	96(15.7%)	124(20.2%)	2.23	1.57–3.1
**Have a condom with you now?**				
Not had condom	107(17.3%)	269(43.5%)	1	–
Showed	92(14.9%)	151(24.4%)	1.53	1.08–2.15
**Does HIV spread through sexual intercourse?**				
No	12(2.7%)	11(2.4%)	1	–
Yes	125(27.8%)	302(67.1%)	2.63	1.13–6.13
**Does HIV spread through sharp**				
**instruments/syringe?**				
No	41(9.1%)	151(33.6%)	1	–
Yes	96(21.3%)	162(36%)	2.18	1.42–3.34
**Does HIV spread through Insect bites?**				
No	119(26.4%)	310(68.9%)	1	–
Yes	18(4%)	4(0.7%)	15.36	4.52–54.03
**Does HIV spread through staying filthy?**				
No	120(26.7%)	305(67.9%)	1	--
Yes	16(3.6%)	8(1.8%)	5.08	2.12–12.18
**Does HIV spread through blood transfusion?**				
No	56(12.4%)	242(53.8%)	1	–
Yes	81(18%)	71(15.8%)	4.93	3.20–7.58
**Does HIV spread through staying filthy?**				
Yes	6(1.3%)	49(10.9%)	1	–
No	129(28.8%)	264(58.9%)	3.99	1.66–9.55
**Prevention is possible by using condoms**				
No	6(1.3%)	177(39.5%)	1	–
Yes	124(27.7%)	141(31.5%)	25.94	11.10–60.1
**Prevention is possible by refraining from sex**				
Yes	55(12.3%)	260(58%)	1	–
No	75(16.7%)	58(12.9%)	6.11	3.90–9.58
**Know where people can go, if they**				
**want to get an HIV/AIDS test?**				
Yes	82(16.3%)	113(22.4%)	1	–
No	85(16.9%)	24(44.4%)	1.91	1.31–2.79
**Ever tested for HIV?**				
Yes	64(12.7%)	8(11.6%)	1	–
No	101(20.1%)	279(55.6%)	3.04	1.99–4.64
				
**Use condom**				
Yes	79(17.3%)	113(24.7%)	1	–
No	69(15.1%)	196(42.9%)	1.98	1.33–2.95
**Wash with Dettol**				
Yes	59(12.9%)	66(14.4%)	1	–
No	89(19.5%)	243(53.2%)	2.44	1.59–3.741
**Take medicine**				
Yes	54(11.8%)	82(17.9%)	1	–
No	94(20.6%)	227(49.7%)	1.59	1.04–2.419
**Arrested in past 6 months**				
Yes	81(13.1%)	41(6.6%)	1	–
No	117(19.0%)	378(61.3%)	6.38	4.15–9.803
**Taken alcohol during sexual act in**				
**the past 6 months?**				
No	88(14.2%)	125(20.2%)	1	–
Yes	111(17.9%	295(47.7%)	3.53	0.37–0.758
				
No	154(25.6%)	396(65.9%)	1	–
Yes	44(7.3%)	7(1.2%)	16.16	7.12–36.16
**Given free condoms in the past one month**				
Yes	118(19.1%)	116(18.7%)	1	–
No	81(13.1%)	304(49.1%)	3.81	2.67–5.443

In univariate analysis, among socio-demographic characteristics factors associated with higher prevalence of HIV in Larkana included younger age 20–24 years (OR: 5.8, CI: 2.81–12.15), being unmarried (OR: 2.4, CI: 1.088–5.717), mode of income only sex work (OR: 5.5, CI: 3.70–8.23) and duration in sex work 5–10 years (OR: 3.3, CI: 1.77–6.12) or more. Those who had to travel outside the city for sex work during last 12 months were significantly more in Larkana than other cities (OR:1.60, CI: 1.13–2.28). In section dealing with *Knowledge and behavior towards HIV and other STI’s* it was found that 16 HSWs in Larkana believed that HIV does spread through kissing, touching or hugging (OR:5.08, CI: 2.12–12.18). More HSWs in Larkana compared to other cities believed that HIV can also spread through blood transfusion (OR: 4.93, CI: 4.93–3.20).

Regarding *Knowledge about prevention of HIV/AIDs* majority of HSWs in Larkana replied *“no” when asked* if prevention is possible by refraining from sex (OR: 6.13, CI: 3.90–9.58). HSWs in Larkana did not know where people could go, if they want to get an HIV/AIDS test (OR: 1.91, CI: 1.31–2.79).

In *section related to practices for prevention of HIV and other STI* significantly low number of HSWs in Larkana used condom during sexual act (OR: 1.98, CI: 1.33–2.95) and many indulged in taking alcohol during sexual act in the past 6 months (OR: 3.53, CI: 0.37–0.75). Comparatively larger number of HSWs in Larkana had sex with an injecting drug user (past 6 months) (OR: 16.163, CI: 7.12–36.16).

Table 5 In multivariate analysis HSWs from Larkana were more likely to lack knowledge regarding preventive measures against HIV (OR 11.9, CI: 3.4–41.08) and were also more prone to use of alcohol during anal intercourse (OR: 6.3, CI: 2.77–17.79).

**Table 5:** Multivariate analysis for the factors associated with high prevalence of HIV among HSWs in Larkana comparing with HSWs in other cities

**Variable**	**OR**	**CI**
*Living with*		
· Others	1	–
· Alone	0.535	0.113–2.535
· Family or relatives	7.452	1.153–48.17
· Friends	1.733	0.321–9.363
*Knowledge about possible ways of HI*		
*V Prevention*		
**Refraining from Sex**		
➢ Yes	1	–
➢ No	11.93	3.4–41.08
**Use of clean syringe**		
➢ Yes	1	–
➢ No	2.99	1.031–8.673
*Alcohol Use during intercourse*		
➢ No	1	–
➢ Yes	6.366	2.77–17.797

## Discussion

The key factors that have been found to be associated with HIV positivity among HSWs in Larkana include younger age, being unmarried, sex work as the only mode of income and longer duration in sex work (5–10 years). Moreover, most of the HSWs in Larkana entertained multiple partners in previous months compared to HSWs from other cities (Table 3). HSWs of Larkana were more likely to lack knowledge regarding preventive measures for HIV. A sizeable proportion of HSWs in our study reported indulging in unprotected anal sex with multiple male partners, both casual and commercial, in the previous month.

Recent studies have shown that high partner turnover along with the lack of knowledge regarding preventive measures has led to a rapid rise in HIV prevalence among sex workers in several Asian cities including Bangkok, China, Indonesia, Nepal and Pakistan [9,11,12]. Pakistan progressed to concentrated level of the epidemic in 2005. During that time four per cent of surveyed male sex workers in Karachi were found to be infected and within two years, that figure had nearly doubled. The results of Round III of surveillance indicated an overall 6.4% HIV among HSWs in the country. The 2008 IBBS data and the analyses undertaken in the present study suggest that HSWs in the country and more so HSWs from Larkana are at increased risk of acquiring HIV infection.

This high prevalence of HIV was alarming. Regional data also indicate that HSWs are at elevated risk for HIV infection in many low and middle income countries of the region like Indonesia, Nepal, Vietnam, China and India [11-17].

The health and social services in Larkana are already fragmented compared to other sites. For example the infant mortality rate of Larkana is 84/1000 live births whereas IMR of Karachi is 40 and Lahore is 50 [18]. More so HSWs are intimidated to seek health services because of the attitude of health care providers.

HSWs in Larkana get engaged in sex work at a relatively early age that increases their vulnerability to HIV. Most probable reason for HSWs to engage in commercial sex in Larkana at an early age is client competition related to the unique feature of the city with a functioning brothel (FSWs) and mini brothels “*musafirkhanas*” where clients can seek services of young boys as well.

They come in commercial sex without prior knowledge of safe behaviors and get infected with HIV compared to HSWs from other parts of the country. Several other measures of sexual risk behavior were also reported more frequently by Larkana HSWs than others.

Even though HSWs in Larkana knew that HIV can spread through blood transfusion but many of them also thought that HIV can also spread through kissing and hugging. They also had quite inadequate knowledge regarding modes of prevention of HIV as in response to the question regarding knowledge of prevention of HIV majority of HSWs in Larkana replied “no” when asked if prevention is possible by refraining from sex (OR: 6.1313, CI: 3.9–9–58). HSWs in Larkana did not know where people could go, if they want to get an HIV/AIDS test (OR: 1.91, CI: 1.31–2.79). It actually indicates lack of access and social barriers to seek help if needed. This is discovered in other global surveys as well that sex workers have inadequate access to HIV prevention services, and even more limited access for appropriate treatment, care and support [11,12]. The epidemiological data on HIV infection rates among sex workers and their clients reflects the failure to adequately respond to their human rights and public health needs. It may also be due to stigma of or being identified as a person at high risk for HIV. While the global response to the pandemic has progressed over the decades both in scale and in efforts to reach diverse and vulnerable groups, stigma and discrimination still follow affected individuals in many settings [17,19-21].

Findings from multivariate analysis showed that the HSWs from Larkana were more prone to use of alcohol during anal intercourse. This is a significant finding but not surprising because drunken HSWs are more likely to ignore safe sex. Literature also points to the use of alcohol and other psycho stimulants as a risk factor for HIV infection among HSWs [22-25]. Substance abuse is also known to be associated with prolonged and rough anal sex, which when combined with reduced condom use, would appear to be a fairly dangerous combination. Increasing consistent condom use is of utmost importance for HIV prevention.

Study findings reinforce the need to develop and implement effective behavioral interventions for HSWs in general and Larkana HSWs in particular in order to reduce transmission risk. Most interventions that target MSM have been developed for “gay” self-identified MSM as well whose sex partners are predominantly other men. However, male clients of these HSWs who have both male and female partners, may not be reached by or respond to such interventions. In Pakistan, locations where “*Hijra* sex workers” work are fairly well established, and it may be feasible to use different strategies and messages. In these settings, it is possible to target sex workers with a full range of risk reduction messages that address both vaginal and anal sex. Moreover evidence based strategies to address the social and financial support needs of sex workers constitute integral component of effective and comprehensive response to HIV. UNAIDS has given a framework based on three pillars to address HIV and sex work i.e.

*Pillar 1:* Assure universal access to comprehensive HIV prevention, treatment, care and support.

*Pillar 2:* Build supportive environments, strengthen partnerships and expand choices.

*Pillar 3:* Reduce vulnerability and address structural issues [26,27].

Working in partnership with sex workers to identify their needs and to advocate for policies and programs that improve their health, safety and engagement in the AIDS response is a proven strategy and an essential feature of UNAIDS approach. Partnerships at national, local and community levels should be strengthened to remove the barriers that sex workers face to service access and their human rights. In addition drivers of the epidemic like social exclusion, stigma and discrimination of HSWs should be addressed. One of the strategies to address could be development and empowerment of transgender community based organizations which exist in negligible number in Pakistan and none in Larkana.

## Conclusion

HIV-related high-risk behaviors were common among HSWs in Larkana, along with a low rate of condom use and multiple sex partners. Poverty, low access to appropriate health care and the limited awareness to HIV spread modes and preventive strategies are the factors that pose a deadly threat for the spread of HIV among vulnerable groups in the country. HSWs are particularly at high risk due to poor access to preventive services and education. They not only suffer physical anguish but also experience isolation, discrimination and abuse.

Hence, HSWs should be the target of specific preventive activities due to their particular vulnerability and the likelihood that the infection may disseminate into the general population given the high proportion of bisexual clients. Comprehensive rights-based programs on HIV and sex work are critical for the success of the HIV prevention efforts and reduction in stigma and discrimination associated with the patients.

## Competing interests

The authors declare that they have no competing interests.

## Authors’ contributions

AA conceived the study and supervised the data collection. AZ and AjA performed the statistical analysis. AA, AZ and AjA drafted and revised the manuscript. All authors read and approved the final draft.

## Pre-publication history

The pre-publication history for this paper can be accessed here:

http://www.biomedcentral.com/1471-2458/12/279/prepub
